# Relationship between dental calcification and skeletal maturation in a Peruvian sample

**DOI:** 10.1590/2177-6709.22.3.089-096.oar

**Published:** 2017

**Authors:** Rocío M. Lecca-Morales, Marcos J. Carruitero

**Affiliations:** 1 Private practice, Trujillo, Peru.; 2 Universidad Privada Antenor Orrego, School of Stomatology, Trujillo, Peru.

**Keywords:** Carpal, Cervical vertebrae, Tooth calcification, Skeletal maturation, Peruvian.

## Abstract

**Objective:**

the objective of the study was to determine the relationship between dental calcification stages and skeletal maturation in a Peruvian sample.

**Methods:**

panoramic, cephalometric and carpal radiographs of 78 patients (34 girls and 44 boys) between 7 and 17 years old (9.90 ± 2.5 years) were evaluated. Stages of tooth calcification of the mandibular canine, first premolar, second premolar, and second molar and the skeletal maturation with a hand-wrist and a cervical vertebrae method were assessed. The relationships between the stages were assessed using Spearman’s correlation coefficient. Additionally, the associations of mandibular and pubertal growth peak stages with tooth calcification were evaluated by Fisher’s exact test.

**Results:**

all teeth showed positive and statistically significant correlations, the highest correlation was between the mandibular second molar calcification stages with hand-wrist maturation stages (*r* = 0.758, *p* < 0.001) and with vertebrae cervical maturation stages (*r* = 0.605, *p* < 0.001). The pubertal growth spurt was found in the G stage of calcification of the second mandibular molar, and the mandibular growth peak was found in the F stage of calcification of the second molar.

**Conclusion:**

there was a positive relationship between dental calcification stages and skeletal maturation stages by hand-wrist and cervical vertebrae methods in the sample studied. Dental calcification stages of the second mandibular molar showed the highest positive correlation with the hand-wrist and cervical vertebrae stages.

## RESUMO

**Objetivo:**

o objetivo do presente estudo foi determinar a correlação entre o estágio de calcificação dentária e a maturação esquelética, em uma amostra de indivíduos peruanos.

**Métodos:**

radiografias panorâmicas, cefalométricas e carpais de 78 pacientes (34 meninas e 44 meninos) com idades entre 7 e 17 anos (média = 9,90 ± 2,5 anos) foram avaliadas. Nelas, avaliaram-se os estágios de calcificação dentária (canino, primeiro pré-molar, segundo pré-molar e segundo molar inferiores) e de maturação esquelética, pelas avaliações radiográficas da mão e punho e das vértebras cervicais. As correlações entre esses estágios foram avaliadas usando-se o coeficiente de correlação de Spearman. Adicionalmente, a associação entre os estágios em que ocorreram os picos de crescimento mandibular e de crescimento puberal e o grau de calcificação dentária foi avaliada pelo teste exato de Fisher.

**Resultados:**

todos os dentes demonstraram correlações positivas e estatisticamente significativas. A correlação mais elevada foi verificada entre o estágio de calcificação do segundo molar inferior e o estágio de maturação esquelética da mão e do punho (*r*= 0,758, *p* < 0,001) e o estágio de maturação das vértebras cervicais (*r* = 0,605, *p* < 0,001). O surto de crescimento puberal foi identificado no estágio G de calcificação do segundo molar inferior, e o pico de crescimento mandibular foi detectado no estágio F de calcificação do segundo molar.

**Conclusão:**

na amostra estudada, houve uma correlação positiva entre os estágios de calcificação dentária e os estágios de maturação esquelética avaliada nas radiografias de mão e punho e das vértebras cervicais. Os estágios de calcificação dentária do segundo molar inferior demonstraram a mais alta correlação positiva com os estágios de maturação da mão e punho e das vértebras cervicais.

## INTRODUCTION

The optimal effectiveness of using braces or orthopedics has been associated with skeletal maturation. Functional appliances have proved to be more effective when used at the peak of mandibular growth, rather than before.^1^ ^,^ ^2^ Therefore, skeletal maturation has been assessed, typically by hand-wrist radiographs,^3^ later on lateral cephalometry^4^ and recently by evaluating the dental calcification of specific teeth on panoramic radiographs.^5^ ^,^ ^6^

For a long time, the growth of the bones of the hand and wrist has been used to assess skeletal maturation,^3^ ^,^ ^7^ ^-^ ^9^ The method described by Fishman^3^ ^,^ ^10^ seems to be the most appropriate method for assessing skeletal maturation.^11^ It considers eleven indicators of skeletal maturation, which cover the entire period of development. However, this method involves exposing the patient to additional radiographic imaging sessions, which is why several researchers^12^ ^-^ ^14^ have developed indexes of skeletal maturation using the profile of cervical vertebrae bodies that appear on lateral radiographs of the skull, which are routinely used for diagnosis in orthodontics. Thus, after several studies,^15^ ^-^ ^18^ Baccetti et al^4^ proposed a method for detecting the peak of mandibular growth by analyzing the second through fourth cervical vertebrae. Although the reproducibility was considered to be poor,^19^ ^,^ ^20^ more recently, visual assessment of the stages with this method has shown acceptable reproducibility and accuracy.^21^

Dental development has been widely investigated as a potential indicator of skeletal maturation.^22^ ^-^ ^26^ Tooth development can be assessed by the stage of calcification,^27^ and it is a very reliable method.^28^ ^,^ ^29^ This method consists of the observation of dental calcification stages to determine the dental maturation of each tooth, while watching the progress of the formation of the crown and root on panoramic radiographs. 

Previous studies^30^ ^-^ ^33^ have evaluated the relationship between skeletal maturation stages and tooth calcification by comparing different methods in different populations. However, there have been no studies reported of this relationship in a sample of Peruvian children despite the fact that there may be differences in the racial background of these children that would need additional investigation. It is also necessary to clarify the discrepancies between the studies because currently it seems unclear^5^ ^,^ ^6^ ^,^ ^34^ ^,^ ^35^ as to which teeth have calcification stages more closely related to skeletal maturity stages, the pubertal growth spurt and the peak of mandibular growth, which could be clarified with measurements performed on the same patients on the same day.

The objective of the present study was to determine the relationships of dental calcification with skeletal maturation in a Peruvian sample. It was hypothesized that there is a positive relationship between dental calcification and skeletal maturation by the methods of hand-wrist and cervical vertebrae in the Peruvian sample studied.

## MATERIAL AND METHODS

### Study sample

The study was conducted with archived panoramic, cephalometric and carpal radiographs of 78 patients (34 girls and 44 boys) between 7 and 17 years old (9.90 ± 2.5 years) in a Stomatology Clinic of Trujillo-Peru (Table 1) who met the selection criteria. The sample was randomly selected using a simple random sample from a total of 702 radiographs (234 cephalometric, 234 panoramic and 234 hand-wrist) obtained from the same patients from 2008 until December 2013. The random selection was made according an excel function [=ALEATORIO.ENTRE(1,702)]. The sample size was calculated using the minor correlation found (0.310) between dental calcification stages and skeletal maturity indicators reported in a previous study.^36^ A statistical power of 80% and a confidence level of 95% were considered. The minimum sample size was 64, however it was decided to increase this by 20% to improve representation. 

**Table 1:** Description of the sample by sex and average age.

Sex	n	%	Average age*	Standard deviation	Range (years)
Male	34	43.6	9.4	2.4	7 - 17
Female	44	56.4	10.3	2.6	7 - 16
Total	78	100	9.9	2.5	7 - 17

*No difference between sex, *t* = -0.89, *p* = 0.188.

Selection criteria were: radiographs (panoramic, cephalometric and carpal radiographs) obtained on the same day and from the same patient, radiographs presenting clear anatomical details, and radiographs with no abnormal dental conditions such as impaction or transposition. The study protocol was approved by a Stomatology Permanent Research Committee of Peru.

### Dental age

To determine the dental age four, left mandibular teeth were evaluated: canine, first premolar, second premolar, and second molar. Dental calcification stages were determined by the Demirjian method (DM),^27^ and each stage was categorized from A to H (Table 2).

**Table 2:** Dental calcification stages using the Demirjian Index method.^27^

Stage	Characteristics
A	Calcification of single occlusal points without fusion of different calcifications.
B	Fusion of mineralization points; the contour of the occlusal surface is recognizable.
C	Enamel formation has been completed at the occlusal surface, and dentin formation has commenced. The pulp chamber is curved, and no pulp horns are visible.
D	Crown formation has been completed to the level of the cement-enamel junction. Root formation has commenced. The pulp horns are beginning to differentiate, but the walls of the pulp chamber remain curve.
E	The root length remains shorter than the crown height. The walls of the pulp chamber are straight, and the pulp horns have become more differentiated than in the previous stage. In the molars, the radicular bifurcation has commenced to calcify.
F	The walls of the pulp chamber now form an isosceles triangle, and the root length is equal to or greater than the crown height. In the molars, the bifurcation has developed sufficiently to give the roots a distinct form.
G	The walls of the root canal are now parallel, but the apical end is partially open. In molars, only the distal root is rated.
H	The root apex is complete.

### Skeletal maturation

Skeletal maturation was evaluated by two methods. The first method was analyzing the hand and wrist, which was performed assessing the six anatomical sites located in the thumb, third finger, fifth finger and radius, according to the parameters set by Fishman.^3^ The hand-wrist method of Fishman (HWMF) details eleven stages (Table 3).The second method was the analysis of the cervical vertebrae, which was performed in the lateral cephalometric radiograph and was analyzed from the second to the fourth cervical vertebrae, according to the stages proposed by Baccetti et al.^4^ The cervical vertebrae method (CVM) determine six stages (Table 4).

**Table 3:** Skeletal maturation indicators (SMI) by the hand-wrist method of Fishman.^3^

Stage	Characteristics
SMI 1	Epiphysis equal in width to diaphysis in the proximal phalanx of the third finger.
SMI 2	Epiphysis equal in width to diaphysis in the middle phalanx of the third finger.
SMI 3	Epiphysis equal in width to diaphysis in the middle phalanx of the fifth finger.
SMI 4	Ossification of adductor sesamoid of thumb.
SMI 5	Capping of epiphysis in the distal phalanx of the third finger.
SMI 6	Capping of epiphysis in the middle phalanx of the third finger.
SMI 7	Capping of epiphysis in the middle phalanx of the fifth finger.
SMI 8	Fusion of epiphysis to diaphysis in the distal phalanx of the third finger.
SMI 9	Fusion of epiphysis to diaphysis in the proximal phalanx of the third finger.
SMI 10	Fusion of epiphysis to diaphysis in the middle phalanx of the third finger.
SMI 11	Fusion of epiphysis to diaphysis in the radius.

**Table 4:** Definitions of cervical stages (CS) using the cervical vertebrae method.^4^

Stage	Characteristics
CS1	The lower borders of all the three vertebrae (C2-C4) are flat. The bodies of both C3 and C4 are trapezoid in shape (the superior border of the vertebral body is tapered from posterior to anterior).
CS2	A concavity is present at the lower border of C2 (in four of five cases, with the remaining subjects still showing CS 1). The bodies of both C3 and C4 are still trapezoid in shape.
CS3	Concavities at the lower borders of both C2 and C3 are present. The bodies of C3 and C4 might be either trapezoid or rectangular and horizontal in shape.
CS4	Concavities at the lower borders of C2, C3, and C4 now are present. The bodies of both C3 and C4 are rectangular and horizontal in shape.
CS5	The concavities at the lower borders of C2, C3, and C4 are still present. At least one of the bodies of C3 and C4 is squared in shape. If not squared, the body of the other cervical vertebra is rectangular and horizontal.
CS6	The concavities at the lower borders of C2, C3, and C4 are still evident. At least one of the bodies of C3 and C4 is rectangular and vertical in shape. If not rectangular and vertical, the body of the other cervical vertebra is squared.

### Errors in the methods

An error of method intra/inter-operator was performed evaluating 10 panoramic radiographs, 10 cephalometric radiographs and 10 carpal radiographs. To determine the concordance in interrater and intrarater measurements, the second observation was after two weeks. Cohen’s unweighted kappa index was used, and the concordances were found to be substantial and almost perfect^37^ with values from 0.733 to 1.000. 

### Statistical analysis

Data was stored and processed in the Stata (StataCorp LP, College Station, Texas, USA) statistical package, version 12. To determine the relationships between the stages studied, Spearman’s correlation coefficient was used. In a second analysis, the skeletal maturation stages were categorized dichotomously as the absence and presence of the pubertal growth spurt in HWMF, considering as presence from the stage 5 to 7^3^ and as absence the previous and subsequent stages. For the peak of mandibular growth in CVM, presence was considered to be on stages 3 to 4^4^ and absence was considered to be the previous and subsequent stages. Fisher’s exact test was used to assess the associations between dichotomized variables and the stages of dental calcification, showing the highest correlations. A significance level of 5% was considered.

## RESULTS

Assessing the relationships of DM stages with HWMF and CVM stages by Spearman’s correlation coefficient, positive and statistically significant correlations were found in canines, premolars and second molars (*p*< 0.001). The strongest correlation between DM stages and HWMF stages was 0.758 and 0.748 with the second molar and second premolar respectively, while between DM stages and CVM stages, the strongest correlation was 0.605 for the same teeth (Table 5).

**Table 5:** Spearman’s correlations among HWMF, CVM and DM (n = 78).

	DM				HWMF	CVM
	Canine	1st PM	2nd PM	2nd M		
HWMF	0.697	0.739	0.748	0.758	1.000	0.817
p	<0.001	<0.001	<0.001	<0.001	<0.001	<0.001
CVM	0.554	0.603	0.605	0.605	0.817	1.000
p	<0.001	<0.001	<0.001	<0.001	<0.001	<0.001

In boys and girls separately, a positive and statistically significant correlation was also found in all of the cases (*p*< 0.001). In boys, the strongest correlation between the stages of DM and HWMF was 0.800 with the second molar; and between DM and CVM, it was 0.684 also with the mandibular second molar. In girls, the strongest correlation between DM and HWMF was 0.792 with the second molar, and between DM and CVM, the strongest correlation was 0.644 with the canine (Table 6).

**Table 6:** Spearman’s correlation among HWMF, CVM and DM by sex.

Sex	DM		CVM		HWMF
		Canine	1st PM	2nd PM	2nd M		
Male (n=34)	HWMF	0.710	0.761	0.784	0.800	0.829	1.000
	p	<0.001	<0.001	<0.001	<0.001	<0.001	<0.001
	CVM	0.578	0.585	0.634	0.684	1.000	0.829
	p	<0.001	<0.001	<0.001	<0.001	<0.001	<0.001
Female (n=44)	HWMF	0.710	0.754	0.759	0.792	0.812	1.000
	p	<0.001	<0.001	<0.001	<0.001	<0.001	<0.001
	CVM	0.542	0.644	0.600	0.534	1.000	0.812
p	<0.001	<0.001	<0.001	<0.001	<0.001	<0.001

Statistically significant associations (*p*= 0.004) between the pubertal growth spurt and calcification stages of the second mandibular molar were found. A higher frequency of subjects in the pubertal spurt growth (HWMF) was found in the G stage. Statistically significant associations (*p*= 0.037) between mandibular growth peak (CVM) and the second mandibular molar calcification stages were also found. The highest frequency of subjects in mandibular growth peak occurred in approximately the F stage. No statistically significant associations (*p*> 0.05) between the pubertal growth spurt and calcification stages of the second mandibular premolar were found (Table 7).

**Table 7:** Associations between pubertal growth spurt/mandibular growth peak and DM stages of mandibular second molar and second premolar (teeth that showed higher correlation).

			DM stages of mandibular second molar														
Teeth	Method	Growth	C		D		E		F		G		H		Total		P
			n	%	n	%	n	%	n	%	n	%	n	%	n	%	
2nd molar	HWMF	Outside the pubertal growth spurt	29	37.2	17	21.8	6	7.7	9	11.5	3	3.8	9	11.5	73	93.6	0.004
		Inside the pubertal growth spurt	1	1.3	0	0.0	1	1.3	0	0.0	3	3.8	0	0.0	5	6.4	
		Total	30	38.5	17	21.8	7	9.0	9	11.5	6	7.7	9	11.5	78	100.0	
	CVM	Outside of mandibular growth peak	25	32.1	15	19.2	4	5.1	4	5.1	3	3.8	8	10.3	59	75.6	0.037
		Inside of mandibular growth peak	5	6.4	2	2.6	3	3.8	5	6.4	3	3.8	1	1.3	19	24.4	
		Total	30	38.5	17	21.8	7	9.0	9	11.5	6	7.7	9	11.5	78	100.0	
2nd premolar	HWMF	Outside the pubertal growth spurt	1	1.3	25	32.1	24	30.8	10	12.8	4	5.1	9	11.5	73	93.6	0.140
		Inside the pubertal growth spurt	0	0.0	1	1.3	0	0.0	1	1.3	1	1.3	2	2.6	5	6.4	
		Total	3	1.3	26	33.3	24	30.8	11	14.1	5	6.4	11	14.1	78	100.0	
	CVM	Outside of mandibular growth peak	1	1.3	23	29.5	17	21.8	7	9.0	2	2.6	9	11.5	59	75.6	0.160
		Inside of mandibular growth peak	0	0.0	3	3.9	7	9.0	4	5.1	3	3.9	2	2.6	19	24.4	
		Total	3	1.3	26	33.3	24	30.8	11	14.1	5	6.4	11	14.1	78	100.0	

## DISCUSSION

The simplification of orthodontic treatment has led to the identification of useful features from routine examination such as for panoramic radiography, which can be used to evaluate the dental calcification and the bone maturation. The present study evaluated the correlations between the stages of DM with the stages of HWMF and CVM in a sample of Peruvian subjects in order to identify if dental calcification is associated with pubertal growth and mandibular growth peak.

The results showed positive correlations consistent with findings reported by previous studies,^30^ ^,^ ^35^ ^,^ ^38^ ^,^ ^39^ however none of these studies assessed the correlations of the DM stages with the HWMF and CVM stages using the same sample at the same time like the present study. The evaluated subjects came from a coastal region from the north of Peru where the population is mostly mixed race with white phenotypic components of Mediterranean (Spanish, Portuguese and Italian) and native-American (Quechua) origin with a mainly swarthy and cinnamon-coloured skin. These characteristics belong to an ethnic group that is found throughout the country but mostly on the coast and less in the mountains and jungle.^40^

High correlation between the stages of the HWMF and DM in the second molar and second premolar was found. Both boys and girls showed highly significant correlation with the second molar. Previous studies^30^ ^,^ ^31^ ^,^ ^41^ ^,^ ^42^ have also found that the stages of dental calcification of the lower second molar showed the strongest correlation with HWMF stages. Current studies^5^ suggest that the lower second molar could be a good reference for determining bone maturation. The use of HWMF has reinforced this finding because this method is considered among the most appropriate for assessing skeletal maturation,^11^ and it also has good reproducibility.^43^ Using the second molar for the analysis of bone maturation might provide an advantage over other teeth because its development tends to continue for a longer period of time, and its apical closure generally extends until 16 years of age.^44^

The strongest correlation between the stages of DM and CVM stages was found in the second molar and second premolar. However, when considering boys and girls separately, this finding was repeated only in boys but not in girls, where the highest correlation was with the first and second premolars. Different results have been reported in other populations.^33^ ^,^ ^45^ ^,^ ^46^ In a population from eastern China^45^ high correlation between the stages of CVM and DM was found in the lower second molar in female subjects and in the lower canine in male subjects; in an Iranian female population^46^ the strongest correlation with the lower lateral incisor was found; in a Polish sample^33^ it was found with the lower second premolars in female subjects and in the lower canines in male subjects. This discordance and variability in the correlations could be explained by the controversial reproducibility of CVM^19^ ^,^ ^20^ or because of racial diversity among the studied populations. 

Further exploratory analysis was performed to identify the stages of tooth calcification that corresponded to the peak of pubertal (for HWMF) or mandibular (for CVM) growth. For this analysis Fisher’s exact test was used because there was at least one expected frequency minor than five, needed for assessing associations^47^ between the qualitative variables generated and the stages of calcification of teeth that showed the highest correlations, the second molar and second premolar. Only significant association with the second molar was found.

The pubertal growth spurt was found in approximately the G stage in the second mandibular molar. The mandibular growth peak was found in approximately the F stage in the second mandibular molar, showing statistically significant associations in both cases. Although these stages include the acceleration, peak and deceleration phases, these results were in some agreement with those reported by Kumar et al.^5^, who found that stages F and G of the second molar corresponded to stages 3 and 4 of the cervical vertebrae method, as proposed by Hassel and Farman.^14^

For the present study, the skeletal maturation was analyzed into two categories, as the presence or absence pubertal and mandibular growth peak. This dichotomization was made because the correlation *per se* does not imply diagnostic accuracy. Similarly, Perinetti et al,^34^ in a cross-sectional study, evaluated the association between dental calcification and skeletal maturation categorizing the skeletal maturation into three categories: pre-pubertal, pubertal, and post-pubertal periods, indicating that the dental calcification was only useful for diagnosing pre-pubertal growth phase. Nevertheless, to demonstrate that dental calcification stages may be used as alternative indicators of skeletal maturation further studies that show sensitivity, specificity, positive predictive value, and positive likelihood ratios in similar populations are necessary. 

This study had the limitation that was a cross-sectional study unable to detect the pubertal peak and the mandibular growth peak. A longitudinal study recording statural heights^3^ or total mandibular lengths (Co-Gn)^4^ respectively should had been developed. Besides, data was analyzed through categorization of presence and absence of the peak of pubertal and mandibular growth, in order to help the analysis. Accurate identification of growth peak is of clinical importance because it determines whether an individual is suitable to undergo orthopedic or surgical treatment; thus, longitudinal studies that evaluate dental calcification are necessary.

In spite of the sample was systematically determined, it could be considered comparatively small, which could be another limitation, but actually it represented a careful selection over 6 years of archiving (2008-2013). In addition, we showed for first time, results in a Peruvian population with mestizo features which are similar to the rest of the country and even to other South American countries.^40^

Identifying skeletal maturation with panoramic radiographs using only one teeth could facilitate decision by the orthodontist as a valid clinical complementary tool for determining the peak of pubertal and mandibular growth, which could reduce, but not replace, the need for carpal radiography. The findings of this study could also be useful for other research purposes.

## CONCLUSION 

A relationship between dental calcification stages of canine, first premolar, second premolar and second molar with the skeletal maturation stages by the methods of hand-wrist and cervical vertebrae in the Peruvian sample studied was found. The dental calcification stages of second molar and the second premolar showed the highest correlations. The pubertal spurt growth was associated with the G stage of the second mandibular molar, and the mandibular growth peak with the F stage. 
